# Glycosaminoglycans analogs from marine invertebrates: structure, biological effects, and potential as new therapeutics

**DOI:** 10.3389/fcimb.2014.00123

**Published:** 2014-09-10

**Authors:** Mauro S. G. Pavão

**Affiliations:** Programa de Glicobiologia, Laboratório de Bioquímica e Biologia Cellular de Glicoconjugados, Instituto de Bioquímica Médica Leopoldo De Meis, Hospital Universitário Clementino Fraga Filho, Universidade Federal do Rio de JaneiroRio de Janeiro, Brazil

**Keywords:** glycosaminoglycans, heparin, dermatan sulfate, selectins, marine invertebrates

## Abstract

In this review, several glycosaminoglycan analogs obtained from different marine invertebrate are reported. The structure, biological activity and mechanism of action of these unique molecules are detailed reviewed and exemplified by experiments *in vitro* and *in vivo*. Among the glycans studied are low-sulfated heparin-like polymers from ascidians, containing significant anticoagulant activity and no bleeding effect; dermatan sulfates with significant neurite outgrowth promoting activity and anti-P-selectin from ascidians, and a unique fucosylated chondroitin sulfate from sea cucumbers, possessing anticoagulant activity after oral administration and high anti P- and L-selectin activities. The therapeutic value and safety of these invertebrate glycans have been extensively proved by several experimental animal models of diseases, including thrombosis, inflammation and metastasis. These invertebrate glycans can be obtained in high concentrations from marine organisms that have been used as a food source for decades, and usually obtained from marine farms in sufficient quantities to be used as starting material for new therapeutics.

## Introduction

Heparin is a highly sulfated glycosaminoglycan composed by disaccharide units containing a hexuronic acid (α-L-iduronic acid or β-D-glucuronic acid) linked 1,4 to α-D-glucosamine. The heparin molecules consist of a heterogeneous mixture of polymers with a similar backbone, which results from variations of sulfation on the D-glucosamine (N-acethylated, N-sulfated, O-sulfated at C6 and/or C3) and on the uronic acid residue (O-sulfated at C2) (Lindahl et al., 1989).

Because of its unique binding to antithrombin, involving a specific pentasaccharide sequence containing a 3-O-sulfated glucosamine, heparin is endowed of a potent anticoagulant activity (Lindahl et al., 1980, 1989). In fact, based on its ability to inhibit fluid phase coagulation, unfractionated heparin (UF) isolated from porcine and bovine intestinal mucosa has been used clinically for decades. However, the therapeutic use of UH is limited mostly by its potent hemorrhagic effect, implying that patients under heparin therapy have to be closely monitored (Hirsh, 1984). UH also has poor bioavailability, requires multiple daily dosing, and has side effects such as heparin-induced thrombocytopenia (Hirsh and Raschke, 2004; Hirsh et al., 2004). To circumvent these problems, different low-molecular-weight heparins (LMW-Hep) have been produced by degrading UF, using a variety of methods, including chemical depolymerization and enzymatic digestion (Hirsh and Raschke, 2004).

In addition to its anticoagulant effect, mammalian UH has also anti-inflammatory effect in several animal models of inflammation, which is possibly mediated by P- and L-selectins. The recruitment of leukocytes from blood and lymphatic systems into tissues facilitates the response to tissue injury. Adhesion molecules of the selectin family (E, P, and L) mediate the initial events that direct the movement of leukocytes across the endothelium in inflamed tissues by interacting with sialylated, fucosylated carbohydrate antigens related to sialyl Lewis^x^ [Sle^x^, Neu5Acα2,3Galβ1,4(Fucα1,3)GlcNAcβ-] found at the cell surface (Lasky, 1995; McEver, 1995; Nelson et al., 1995; Butcher and Picker, 1996). It has been reported (Stevenson et al., 2005) that the dose of UH required for the inhibitory effect on the selectins is higher than that required for the anticoagulant action, which increases the hemorrhagic risk and makes the clinical use of UH impractical to treat inflammation. Similarly, the use of LMW-Hep, which has a much lower hemorrhagic effect, is not a good alternative for UH, since it is a poor inhibitor of selectins (Stevenson et al., 2005).

UF and heparin-like oligosaccharides inhibit L- and P-selectin binding to Sle^x^ and has been shown to dramatically reduce leukocyte infiltration in thioglycollate-induced peritoneal inflammation in mice (Burg et al., 1997; Wang et al., 2002). In addition, UH has also been used successfully as a therapeutic agent in different animal models of nephropathy. Subcutaneous injection of non-anticoagulant UH reduces glomerulosclerosis in rats (Burg et al., 1997), and ameliorates the progression of renal disease in rats with subtotal renal ablation (Purkerson et al., 1988). In addition, it has also been demonstrated that heparin inhibits macrophage infiltration and TGF-β synthesis in puromycin glomerulosclerosis (Ceol et al., 2003).

The risk of contamination with pathogens is an important aspect to take into account in the therapeutic use of natural products from mammalian origin. For example, the association of mammalian prionic proteins with transmissible spongiform encephalopathy has restricted the use of bovine heparin in Europe, USA and Japan. In these countries, commercial heparin is obtained exclusively from porcine tissues and the risk of contamination with a prionic protein or even a virus is still present.

Therefore, as we consider the molecular mechanism of the anti-inflammatory effect of UF, its side effects, and the possibility of pathogen contamination, it becomes extremely relevant the search for alternative heparin-like compounds, obtained from non-mammalian sources, possessing similar biological activities, but devoid of the undesired side effects.

## Unique invertebrate glycosaminoglycans

### Heparin-like glycans

Heparin with a structure similar to that of vertebrate heparin but with lower anticoagulant activity has been identified in the tissues of the ascidian *Styela plicata* (Chordata-Tunicata) (Cavalcante et al., 2000). An extensive structural analysis of the polymer indicated that the invertebrate heparin is composed mainly of the disaccharide α-L-IdoA(2SO_4_)-1→4β-D-GlcN(SO_4_)(6SO_4_)-1, with a minor contribution (~25%) of the disaccharide α-L-IdoA-1→4β-D-GlcN(SO_4_)(6SO_4_)-1. Activated partial thromboplastin time (APTT) assays of the ascidian heparin showed that the polymer has lower anticoagulant activity than mammalian heparin. In addition, it is about 20 times less potent than mammalian heparin in stimulating the inhibition of thrombin by purified ATIII. However, *S. plicata* heparin activates HCII with approximately the same potency as vertebrate heparin, as indicated by comparison of the IC_50_ values for inhibition of thrombin by purified HCII. To compare the hemorrhagic effect of the ascidian and mammalian heparin, we used a rat cut-tail bleeding model. *S. plicata* heparin at a dose of 4 mg/kg body weight, which is above the dose required to prevent thrombus on an animal experimental model did not increase the amount of blood loss in comparison with the saline control. In contrast, mammalian heparin, compared to the control, increased blood loss almost 2-fold (Cardilo-Reis et al., 2006).

In a TNBS-induced colitis model in rats, *S. plicata* heparin drastically reduced inflammation after subcutaneous administration during a 7-day period (Belmiro et al., 2009), as observed by the normalization of the macroscopic and histological characteristics of the colon. At molecular level, TNF-alpha, TGF-beta, and VEGF were reduced to normal values. Lymphocyte and macrophage recruitment and epithelial cell apoptosis were also decreased after the treatment. A drastic reduction in collagen-mediated fibrosis was also observed. No hemorrhagic events were observed after glycan treatment (Belmiro et al., 2009). These results strongly indicate a potential therapeutic use of the ascidian heparin in the treatment of colonic inflammation with a lower risk of hemorrhage, when compared with mammalian heparin.

The bivalve mollusk *Nodipecten nodosus*, currently cultivated in different parts of the world with an annual production of about 75,000 tons (http://www.fao.org/fishery/topic/14884/en), has been shown to contain high amounts of a heparan sulfate-like glycosaminoglycan. 1D 1^H^, 2D COZY and HSQC nuclear magnetic resonance revealed characteristic signals of non-sulfated, 3- or 2-sulfated glucuronic acid, as well as *N*-sulfated and/or 6-sulfated glucosamine (Gomes et al., 2010). The mollusk glycan possesses an anticoagulant activity of 36 IU mg^−1^, 5-fold lower than bovine heparin (180 IU mg^−1^). It inhibits factor Xa (IC_50_ = 0.835 μg mL^−1^) and thrombin (IC_50_ = 9.3 μg mL^−1^) in the presence of antithrombin. Experiments *in vivo*, demonstrated that, the mollusk HS inhibited thrombus growth in photochemicaly injured arteries, at the dose of 1 mg Kg^−1^. No bleeding effect, factor XIIa-mediated kallikrein activity or toxic effect on fibroblast cells were induced by the invertebrate HS at the antithrombotic dose (Gomes et al., 2010).

### Oversulfated dermatans

The ascidians *S. plicata* and *Halocyntia pyriformis*, contain dermatan sulfates formed by [α-L-IdoA(2SO_4_)-1→3β-D-GalNAc(4SO_4_)] disaccharide units. These oversulfated dermatans have high heparin cofactor II-mediated anticoagulant activity (Pavao et al., 1998). Due to a higher concentration of [α-L-IdoA(2SO_4_)-1→3β-D-GalNAc(4SO_4_)]-containing sequences, that bind to the glycosaminoglycan binding site in the inhibitor, their heparin cofactor II activities are at least 10 times higher than that of the mammalian counterpart.

The occurrence of a well-defined relationship between sulfate position on the disaccharides and biological activity can be observed studying dermatan sulfates from different species of ascidians. In the ascidian *Phallusia nigra*, the dermatan sulfate has the same degree of sulfation of that from *S. plicata*, but is composed mainly by [α-L-IdoA(2SO_4_)-1→3β-D-GalNAc(6SO_4_)] disaccharide units (Pavao et al., 1995). As a result of the different sulfation on the N-acetylgalactosamine (6-sulfated instead of 4-sulfated), *P. nigra* dermatan sulfate has very low heparin cofactor II-mediated anticoagulant activity. Overall, these results strongly suggest that binding of oversulfated dermatan sulfate polymers to heparin cofactor II requires a specific sulfation pattern on the glycans, composed by [α-L-IdoA(2SO_4_)-1→3β-D-GalNAc(4SO_4_)]-enriched sequences.

These unique oversulfated dermatan sulfates from ascidians, with different heparin cofactor II activities, allowed the study of the relationship between heparin cofactor II activity and antithrombotic effect. Thus, after intravascular administration *S. plicata* dermatan sulfate, with high heparin cofactor II activity, prevents thrombus formation in veins (Vicente et al., 2001). On the other hand, *P. nigra* dermatan sulfate, with a discernible heparin cofactor II activity, has no antithrombotic effect in the same venous thrombotic model and dose (Vicente et al., 2001). These results indicate that a heparin cofactor II-mediated mechanism is associated with the antithrombotic effect of dermatan sulfate polymers.

The oversulfated dermatan sulfates from the ascidian *P. nigra* and *S. plicata*, composed by [α-L-IdoA(2SO_4_)-1→3β-D-GalNAc(6SO_4_)] and [α-L-IdoA(2SO_4_)-1→3β-D-GalNAc(4SO_4_)] disaccharide unities were used in the study of mouse hippocampal neurons behavior. *P. nigra* dermatan possesses significant neurite outgrowth-promoting activity, which resulted specific morphological features. The ascidian dermatan sulfate induced a flattened neuronal cell soma and dendrite-like multiple neurites (Hikino et al., 2003). *S. plicata* dermatan sulfate, composed by [α-L-IdoA(2SO_4_)-1→3β-D-GalNAc(4SO_4_)], on the other hand, exhibited only a modest neurite outgrowth-promoting activity (Hikino et al., 2003; Bao et al., 2005).

Heparin has been shown to exhibit P- and/or L-selectin-mediated antimetastatic and antiinflammatory activities. P-selectin-mediated platelet-tumor cell and tumor-cell endothelium interactions facilitate the initial steps of hematogeneous metastasis (Borsig et al., 2001, 2002). The dermatan sulfates from *S. plicata* and *P. nigra*, that contain the same disaccharide core structure [α-L-IdoA(2SO_4_)-1→3β-D-GalNAc]_n_, but sulfated at carbon 4 or 6 of the GalNAc residues, respectively, and opposed HCII activities are potent inhibitors of P-selectin (Kozlowski and Pavao, 2011; Kozlowski et al., 2011). Both ascidian dermatan sulfates regardless of the position of sulfation on the N-acetylgalactosamine drastically attenuate metastasis of both MC-38 colon carcinoma and B16-BL6 melanoma cells, and the infiltration of inflammatory cells in a thioglycollate peritonitis mouse model (Kozlowski and Pavao, 2011; Kozlowski et al., 2011). Additionally, both ascidians glycosaminoglycans reduced thrombus size in a FeCl_3_-induced arterial thrombosis model, irrespective of their HCII activities. Interestingly, the arterial thrombi demonstrated a markedly reduced platelet deposition after dermatan sulfate treatment (Kozlowski and Pavao, 2011; Kozlowski et al., 2011), suggesting that the ascidians glycosaminoglycan inhibited P-selectin and thereby the binding of activated platelets during thrombus formation. These results provide evidence that inhibition of P-selectin is a potential therapeutic target in thrombosis, inflammation and metastasis, and ascidian dermatan sulfates may serve as anti-selectin agents.

### Fucosylated chondrotitin sulfate

A unique natural chondroitin sulfate analog, containing sulfated fucose branches has been identified in several species of sea cucumbers (Vieira and Mourao, 1988; Vieira et al., 1991; Kariya et al., 1997). The *Ludwigothurea grisea* glycan has a core like that of mammalian chondroitin sulfate but substituted at the 3-position of the glucuronic acid residues with fucose-2,4 disulfated branches (Vieira and Mourao, 1988; Vieira et al., 1991). The fucosylated chondroitin sulfate has high anticoagulant and antithrombotic activities that disappear after removal of the sulfated fucose branches by mild acid hydrolysis (Mourao et al., 1996). Two anticoagulant mechanism have been proposed: activation of thrombin inhibition by heparin cofactor II, and inhibition of factor-Xa and thrombin generation by the tenase and prothrombinase complexes, respectively (Glauser et al., 2008; Buyue and Sheehan, 2009).

Interestingly, thrombosis inhibition in artery by the fucosylated chondroitin sulfate occurs at low doses, and does not modify the plasma anticoagulant activity. On the contrary, in venous thrombosis the antithrombotic activity of the fucosylated chondroitin requires high doses and occurs with an increase in the plasma anticoagulant activity (Zancan and Mourao, 2004). Additionally, daily oral doses of this glycosaminoglycan showed a decrease in thrombus weight on experimental models of venous and arterial thrombosis in experimental animals (Fonseca and Mourao, 2006).

The fucosylated chondroitin sulfate from *H. grisea* inhibited P- and L-selectin binding to immobilized sialyl Lewis^x^, a component of leukocyte surface glycoproteins, which is also overexpressed in several tumor cells (Borsig et al., 2007). The glycan also inhibited LS180 carcinoma cell attachment to immobilized P- and L-selectins (Borsig et al., 2007). As a result of its anti-selectin effect, the intact sea cucumber glycan attenuates lung colonization by adenocarcinoma MC-38 cells in an experimental metastasis model in mice, as well as neutrophil recruitment in thioglycollate-induced peritonitis. Removal of the sulfated fucose branches abolishes the inhibitory effect *in vitro* and *in vivo* (Borsig et al., 2007). These results suggest that this glycan may be a potential therapeutic drug for blocking metastasis and inflammatory reactions.

## Concluding remarks and future perspectives

Glycosaminoglycans analogs with unique structures and pharmacological activities have been described in different marine invertebrates (Table 1). The structure, biological activity and mechanism of action have been extensively studied and the glycans evaluated in pre-clinical experiments in rodent animals with promising results. Heparin-like polymers with low anticoagulant activity, significant antithrombotic and anti-inflammatory activities, but devoid of bleeding effects occur in different species of ascidians and mollusks. Similarly, oversulfated dermatan sulfates containing the same disaccharide core structure of [α-L-IdoA(2SO_4_)-1→3β-D-GalNAc]_n_, but differing in the position of sulfation (4-sulfate or 6-sulfate) on the N-acetylgalactosamine, occur in high amounts in ascidians. These polymers have high anti-selectin activity, which results in attenuation of metastasis and leukocyte recruitment. The 2,6-sulfated dermatan sulfate from *P. nigra* has very low heparin cofactor II activity and antithrombotic effect, different from its 2,4-sulfated high anticoagulant counterpart from *S. plicata*. The anti-P-selectin activity of these disulfated glycans is involved in low platelet arterial thrombus formation. Moreover a significant neurite outgrowth promoting activity is associated with the di-sulfated ascidian dermatan sulfates. The fucosylated chondroitin sulfate from *L.grisea* has significant therapeutic effect after oral administration, attenuating metastasis and inflammation due to the high anti-selectin activity of the sulfated fucose branches. So far, no significant toxic effect has been associated with the use of these marine compounds. The marine invertebrates that are the primary source of these potential therapeutic compounds abound in different parts of the world, mainly in western seas, where they are cultivated in large scale. In general, the marine invertebrate glycans occur in higher concentration in the tissue (about 0.5% of the dry weight, comparing to 0.022% from pig intestinal mucosa) and can be easily isolated by procedures similar to those already employed in the preparation of pharmaceutical heparin. Whereas these marine organisms will be a source of new heparin analogs with significant therapeutic effect in thrombosis, inflammation and cancer in the future will depend on the economic pressure of the pharmaceutical industry and the increasing demand for new natural drugs with less undesired side effects to treat specific diseases.

**Table 1:** **Effect of invertebrate glycans on coagulation, inflammation, and metastasis**.

**Glycan**	**Disaccharide composition**	**Marine invertebrate source**	**Anticoagulant properties**					**Anti P-selectin-mediated events**		
			**aPTT (U/mg)**	**IC_50_ for IIa inhibition (μg/ ml)**		**Effect on thrombosis**	**Bleeding effect (rat tail)**	**Inhibition of tumor cell-selectin adhesion (μg/ml)**	**Inhibition of neutrophil recruit-ment on Tioglycolate-induced peritonitis (%)**	**Inhibition of carcinoma cell metastasis to the lungs (%)**
				**Protease inhibitor**		**Dose for 100% inhibition (mg/kg)**				
		**Ascidian**		**AT**	**HCII**					
Dermatan sulfate	IdoA**2**S-GalNAc**4**S	*S. plicata*	8	ND	0.31	4	Low	13.51^***^	>90^**^	>95^***^
Dermatan sulfate	IdoA**2**S-GalNAc**6**S	*P. nigra*	<0.5	ND	320	>400	Low	12.19^***^	>90^**^	>95^***^
		**Bivalve mollusk**								
Heparan sulfate-like	**GlcA-GlcNAc** (GlcA**2**S/GlcA**3**S) (Glc**N**S/GlcNAc**6**S)	*N. nodosus*	38.3	0.012	4	1	Low	0.07^****^	>90^*****^	~50^****^
		**Sea cucumber**								
Fucosylated chondroitin sulfate	(Fuc**2**/**3**S)-GlcA-GalNAc**6**S	*L. grisea*	40	~9^⨛^	~1^⨛^	3	Low	10.4^***^	~80^*^	>95^***^

* *Dose of 0.5 mg/mouse, see Borsig et al. (2007)*.

** *Dose of 0.1 mg/mouse, see Kozlowski et al. (2011)*.

*** *Carcinoma MC-38 cell, see Kozlowski et al. (2011)*.

**** *Carcinoma LS 180 cell, unpublished results*.

***** *Dose of 0.5 mg/mouse, unpublished results*.

⨛ *See reference Mourao et al. (1996)*.

### Conflict of interest statement

The author declares that the research was conducted in the absence of any commercial or financial relationships that could be construed as a potential conflict of interest.

## Abbreviations

HexA: hexuronic acid
GlcA: glucuronic acid
IdoA: iduronic acid
GalNAc: N-acetyl galactosamine
GlcNAc: N-acetyl glucosamine
GlcA2S: 2-O-sulfated glucuronic acid
GlcA3S: 3-O-sulfated glucuronic acid
IdoA2S: 2-O-sulfated iduronic acid
GalNAc4S: 4-O-sulfated N-acetyl galactosamine
GalNAc6S: 6-O-sulfated N-acetyl galactosamine
GalNAc4S,6S: 4-O- and 6-Osulfated N-acetyl galactosamine
GlcNS: N-sulfated glucosamine
GlcNS,6S: N- and 6-sulfated glucosamine
GlcNS,3S,6S: N, 3-O- and 6-Osulfated glucosamine.
